# Anesthetic management of caesarean section of a pregnant woman with cerebral arteriovenous malformation: a case report

**DOI:** 10.1186/1757-1626-1-327

**Published:** 2008-11-18

**Authors:** Demet Coskun, Ahmet Mahli, Zerrin Yilmaz, Pelin Cizmeci

**Affiliations:** 1 Department of Anesthesiology and Reanimation Gazi University Faculty of Medicine, Ankara, Turkey

## Abstract

**Introduction:**

The choice of anesthetic technique for Caesarean section of a pregnant woman with cerebral arteriovenous malformation (AVM) is made to maintain a stable cardiovascular system, but due to the rarity of this condition, no definitive guidelines exist.

**Case Presentation:**

We report the case of anesthetic management of Caesarean section of a pregnant woman with cerebral AVM (grade V). After the diagnosis, the radiologists decided to perform angiography and endovascular operation for treatment after the termination of pregnancy. The patient refused to undergo this procedure and with the beginning of the contractions of uterus, she was admitted to hospital urgently at the 40^th ^week of gestational age and Caesarean section under general anesthesia was performed successfully.

**Conclusion:**

We concluded that in case of emergency, general anesthesia can be used satisfactorily for Caesarean section of a pregnant woman with cerebral AVM. Ensuring optimal maternal and fetal well-being, we are of the opinion that it is also possible to control the arterial blood pressure of patients with general anesthesia.

## Introduction

Cerebrovascular diseases are quite rare in pregnancy [1]. Cerebral arteriovenous malformations (AVM) are present in approximately 1:10.000 of the population and are responsible for approximately 10% of subarachnoid hemorrhages in general population [2]. Intracranial hemorrhage due to rupture of an AVM during pregnancy is a rare but serious condition; when it occurs, both the mother's and the fetus's well-being is effected [3].

The choice of anesthetic technique for Caesarean section of these patients is made to maintain a stable cardiovascular system [4], but due to the rarity of this condition, no definitive guidelines exist. Anesthetic considerations during pregnancy are governed primarily by maternal-fetal physiology and arterial pressure control to reduce the risk of aneurismal hemorrhage. Participation of perinatal anesthesiologists experienced in obstetric anesthesia is desirable [5].

## Case presentation

We report the case of anesthetic management of Caesarean section of a pregnant woman with AVM. A 24-year-old primipara at the 16^th ^week of gestational age was admitted the the hospital with a sudden severe headache without any complaint of nausea or vomiting. A cerebral magnetic resonance imaging revealed giant (grade V) cerebrothalamic AVM. The radiologists decided to perform angiography and endovascular operation for treatment after the termination of pregnancy; however, the patient did not allow the termination of pregnancy. The treatment of AVM was postponed until after the end of pregnancy. There were no further problems during the rest of the pregnancy. The patient was admitted to the hospital at the 40^th ^week of gestational age urgently with uterine contractions and Caesarean section under general anesthesia was performed. Maternal monitoring consisted of invasive arterial blood pressure, in addition to the standard monitoring for anesthesia; mean arterial pressure (MAP), heart rate, peripheric O_2 _saturation (SpO_2_) values measured before the induction, for every 2 minutes after the induction until the extubation and for every 5 minutes in the recovery room for 30 minutes. Anesthetic care was directed at ensuring optimal maternal and fetal well-being, and was induced with propofol (2 mg kg^-1^), succinylcholine (1 mg kg^-1^) and remifentanil (0.1 μg kg^-1^min^-1^) was administered to facilitate tracheal intubation, and intravenous lidocaine (1 mg kg^-1^) and nitroglycerin (0.5 μg kg^-1^min^-1^) were used to reduce the hypertensive response to tracheal intubation. Anesthesia was maintained with propofol until the delivery of the infant. The Apgar score was 8/9 at the 1^st ^and the 5^th ^minutes. After the delivery, anesthesia was maintained with rocuronium (0.3 mg kg^-1^), remifentanil (0.1–0.2 μg kg^-1^min^-1^) and propofol (4–8 mg kg^-1^hour^-1^) infusion with air/O_2 _(%50-50). We also infused nitroglycerin continuously at a rate of 0.25–0.5 μg kg^-1^min^-1 ^to control arterial blood pressure due to invasive arterial blood pressure monitoring (MAP approximately 70 mm Hg). The patient was mechanically ventilated to maintain mild hypocapnia (end-expiratory CO_2 _approximately 33 mmHg). Following the completion of surgical procedures, the patient was extubated before being awakened. Her ventilation was assisted via a mask until she was able to breathe without any support. The patient promptly emerged from anesthesia and was neurologically normal in the operating room and postanesthesia care unit. One week later, angiography and endovascular operation were performed successfully and than the patient was discharged in good condition.

## Discussion

The time of hemorrhage due to AVM in pregnant women is most common at the 15^th^-20^th ^weeks in younger patients but bleeding may ocur at any stage including during labour or in the puerperium [6]. In our case, the patient was symptomatic at the 16^th ^week of gestation. The uterine contractions of labour and the Valsalva effect of vaginal delivery are accompanied by dramatic, transient increases in venous pressure, cardiac output, and cerebrospinal fluid pressure. For this reason, Caesarean section is recommended for patients with inoperable AVMs [3].

Successful treatment requires thorough diagnostics and close monitoring in a flexible teamwork to address both the varying maternal and fetal needs. The fundamental aims of anesthesia are to maintain oxygenation and stable systemic, cerebral and placental hemodynamics and to avoid increased intracranial pressure [7].

The most important problem in anesthetic management for the pregnant that has an emergency Caesarean section and AVM is the acute subarachnoid hemorrhage due to the intra-operative rupture caused by hypertension. So far various volatile anesthetics, regional anesthetic techniques and antihypertensive agents have been used. However, it is impossible to select one of these methods as precisely superior to the others since most of the studies were merely case presentations. Anesthetic goals for these patients included fetal and maternal well being.

The regional anesthetic technique could be preferred because it avoids the haemodynamic stress associated with laryngoscopy, intubation and extubation during general anesthesia [4,8] but in this case, because of the emergency of the case, we preferred general anesthesia and haemodynamic stress was prevented by using nitroglycerin, remifentanil, lidocaine and propofol during induction. During the maintenance of anesthesia, an invasive monitorization of arterial blood pressure was used to prevent hypertension. We maintained both the intra-cranial pressure and the uteroplacental perfusion at appropriate levels by keeping the mean arterial pressure within certain limits.

It is known that preoperative maternal hypothermia appears to be well tolerated by the fetus but the use of hypotensive techniques is more controversial. It is suggested that the fetus is kept protected from hypotension induced by the careful use of volatile anesthetics, beta-adrenoreceptor blockers or sodium nitroprusside. It is also important to avoid hypotension during epidural block, and the increase in intracranial pressure caused by vomiting can be dangerous [8]. The securing of the aneurysm, such as balancing the treatment of undesired hyper/hypotension with the risk of possible re-bleeding/cerebral hypoperfusion, allows greater flexibility in managing the remainder of the pregnancy. Furthermore, Caesarean section can be undertaken without any concern for the fluctuating cerebral perfusion pressure that accompanies Caesarean section [9]. For these reasons, we preferred general anesthesia to keep the mean arterial pressure within certain limits. Additionally, we also have to take into consideration the possible side effects of the chosen anesthetic agent on the fetus.

In recent times; remifentanil is a synthetic opiate with evident advantages for various anesthetic techniques, enhancing the quality of anesthesia. Remifentanil may be used in obstetric analgesia-anesthesia thanks to the advantages demonstrated in patients with heart disease and in those requiring neuron-anesthesia. Remifentanil is known to cross the placenta rapidly and to be rapidly metabolized and redistributed to both the mother and the fetus. Based on this, and on pharmacokinetic and pharmacodynamic studies on children [10], we judged remifentanil to be indicated for use for our patient who underwent an emergency Cesarean section and for whom hemodynamic stability and immediate postoperative assessment were basic requirements.

We concluded that general anesthesia can be used satisfactorily for the Caesarean section of a pregnant woman with cerebral AVM in case of emergency. Ensuring optimal maternal and fetal well-being, we are of the opinion that it is also possible to control the arterial blood pressure of patients with general anesthesia.

## Consent

Written informed consent was obtained from the patient for publication of this case report and accompanying images. A copy of the written consent is available for review by the Editor-in-Chief of this journal.

## Competing interests

The authors declare that they have no competing interests.

## Authors' contributions

DC and AM presented the case history, researched the topic and helped draft the manuscript. ZY and PC reviewed the literature and drafted the manuscript. All authors read and approved the final manuscript.
